# Simultaneous Bilateral Reconstruction of Chronic Achilles Tendon Rupture with Flexor Digitorum Longus Transfer and Turndown Flaps: A Case Report and Review of Literature

**DOI:** 10.3390/jcm15030922

**Published:** 2026-01-23

**Authors:** Simone Daniel Gatti, Carlo Dante Maria Conti, Agostino Dario Caminita, Judith Waldner, Marco Turati, Giovanni Zatti

**Affiliations:** 1 Orthopedic Department, IRCCS San Gerardo dei Tentori, 20900 Monza, Italyagostinodario.caminita@irccs-sangerardo.it (A.D.C.); j.waldner@campus.unimib.it (J.W.); marco.turati@unimib.it (M.T.); giovanni.zatti@unimib.it (G.Z.); 2 School of Medicine and Surgery, University of Milano-Bicocca, 20900 Monza, Italy

**Keywords:** chronic Achilles tendon rupture, bilateral reconstruction, tendon transfer, flexor digitorum longus (FDL), flexor hallucis longus (FHL), Turndown flap augmentation

## Abstract

**Background/Objectives:** Bilateral Achilles tendon ruptures are exceptionally rare, accounting for <1% of Achilles injuries; chronic presentations are frequently linked to diagnostic delay in the acute phase. The aim of this paper is to describe a modified, single-stage bilateral reconstruction of neglected Achilles tendon ruptures using Flexor Digitorum Longus (FDL) transfer and turndown flaps. This study evaluates 12-month functional and radiological outcomes while comparing this surgical strategy with current literature. **Methods**: A 58-year-old man with rheumatoid arthritis on long-term corticosteroids presented 4 months after asynchronous bilateral Achilles ruptures with progressive weakness and gait limitation. Clinical examination and MRI confirmed chronic discontinuity with retracted stumps and interstump gaps of ~6.5–7.0 cm. Intervention: After pre-operative tapering of corticosteroids, a single-stage bilateral reconstruction was performed. Surgical intervention involved simultaneous bilateral reconstruction using flexor digitorum longus (FDL) transfer for one tendon and a combination of FDL and flexor hallucis longus (FHL) transfers for the other, with bilateral turndown flap augmentation. Decisions regarding tendon transfers were based on intraoperative findings, with the FDL selected for its larger caliber when the FHL was deemed insufficient. **Results**: Recovery was uneventful. At 6 months, the patient resumed full-time work and could perform repeated tiptoe rises. The AOFAS ankle-hindfoot score improved from 46 pre-operatively to 85. At 12 months, MRI demonstrated bilateral tendon continuity without re-rupture, with hypertrophy at the reconstructed stumps. **Conclusions**: In chronic, large-gap bilateral Achilles ruptures with systemic risk factors, single-stage reconstruction using FDL (with or without FHL) plus turndown augmentation is feasible and yields favorable functional recovery. Careful tensioning, secure calcaneal fixation, steroid optimization, and structured rehabilitation appear pivotal to outcomes. This case supports the applicability of this strategy in complex bilateral presentations.

## 1. Introduction

Bilateral Achilles tendon ruptures are exceptionally rare, accounting for less than 1% of all Achilles injuries. Predisposing factors include systemic conditions such as rheumatoid arthritis (RA), long-term corticosteroid therapy, and fluoroquinolone use [1,2]. Patients with RA are at an elevated risk due to chronic inflammation, tendon degeneration, and the adverse effects of glucocorticoids on collagen synthesis and vascularization [3]. A reported 20–25% of acute ruptures may be missed and lead to late diagnosis. When left untreated, chronic ruptures develop within 4 to 6 weeks, characterized by tendon retraction, scarring and muscle atrophy, leading to the inability to perform direct end-to-end repair. The optimal surgical strategy remains debated, particularly in cases involving delayed presentations or bilateral injuries.

Various techniques have been described for managing chronic Achilles ruptures, including tendon transfers and biological augmentations. Turndown flaps or V-Y advancement flaps are frequently employed in cases of significant retraction to restore tendon length and improve repair strength. The choice of donor tendons is another critical aspect of surgical decision-making. Flexor hallucis longus (FHL) transfer is widely considered the gold standard due to its anatomical alignment, biomechanical efficiency, and low donor-site morbidity. However, flexor digitorum longus (FDL) transfer has emerged as an alternative, particularly in cases where larger tendon volume is required. FDL is noted for providing additional strength in larger defects while preserving hallux function, making it an attractive choice in select cases. In this report, the FDL transfer was preferred on one side due to its greater tendon caliber, which was approximately double that of the FHL, ensuring better graft-to-defect matching.

Aim: To present a modified surgical strategy for the single-stage, simultaneous bilateral reconstruction of neglected Achilles tendon ruptures in a patient with rheumatoid arthritis. This study details the intraoperative decision-making process for utilizing Flexor Digitorum Longus (FDL) transfer augmented by bilateral turndown flaps. We evaluate clinical success through 12-month functional outcomes (AOFAS scores) and MRI evidence of tendon continuity, while contextualizing this approach within a comprehensive review of existing surgical literature and current evidence-based guidelines.

## 2. Case Presentation

### 2.1. Patient Information

A 58-year-old male truck driver, presented with bilateral chronic Achilles tendon ruptures. The injuries occurred while descending from his truck, two weeks apart. The patient’s medical history included RA treated with hydroxychloroquine and low-dose prednisone (5 mg/day). He presented four months post-injury, reporting significant difficulty ambulating and requiring assistive devices. Initial diagnostic workup included ultrasounds, as the clinical presentation raised suspicion of deep vein thrombosis (DVT). However, magnetic resonance imaging (MRI), performed only after a prolonged delay, ultimately confirmed chronic bilateral Achilles tendon ruptures.

In preparation for surgery, corticosteroid therapy was gradually tapered under rheumatologic guidance and fully discontinued in the immediate postoperative period.

### 2.2. Clinical Examination and Imaging

**Figure 1:**
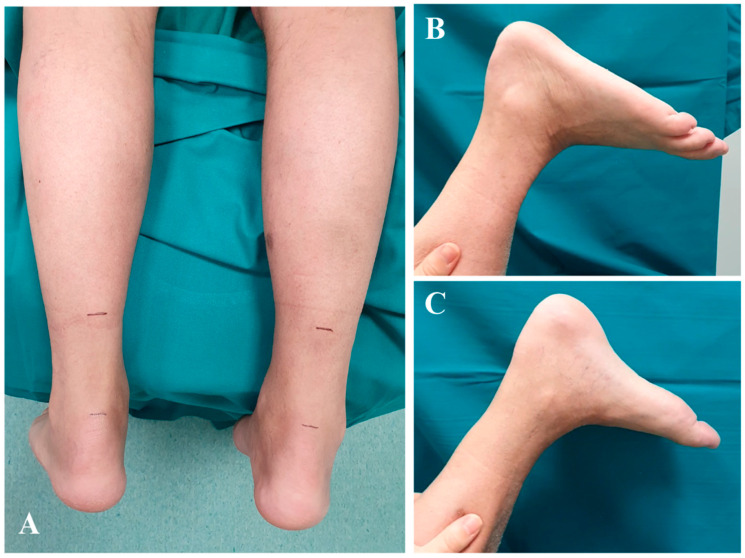
(**A**) Clinical images with bilateral palpable gaps and positive Thompson test ((**B**) left foot; (**C**) right foot).

**Figure 2:**
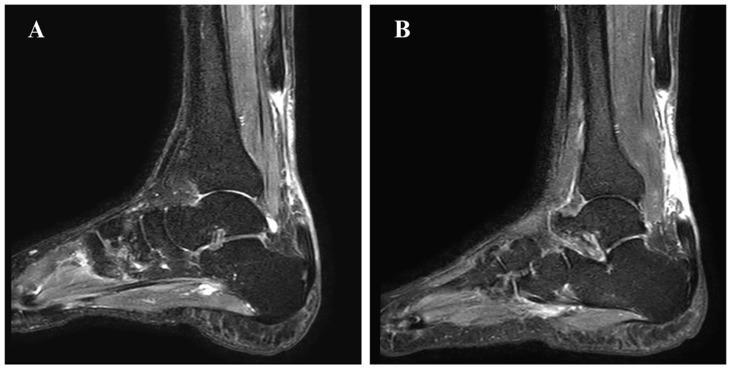
Pre-operative MRI (T2-weighted) revealing tendon rupture with significant retraction ((**A**) left foot 7.0 cm gap; (**B**) right foot 7.5 cm gap).

Physical examination revealed significant hypotrophy of the gastrocnemius-soleus complex, palpable gaps approximately 3–4 cm proximal to the calcaneal insertions, increased dorsiflexion and positive Thompson tests bilaterally (Figure 1). The preoperative American Orthopaedic Foot & Ankle Society (AOFAS) score was 46, reflecting significant functional impairment. Magnetic resonance imaging (MRI) confirmed bilateral chronic Achilles tendon ruptures with retracted stumps and a gap measuring 6.5 cm on the right and 7 cm on the left (Figure 2).

### 2.3. Surgical Approach

A single-stage bilateral reconstruction was performed to optimize recovery and minimize anesthesia exposure. Throughout the procedure, meticulous care was taken to respect the soft tissues and minimize postoperative complications. No surgical forceps were used on the skin; only atraumatic anatomical forceps were employed to handle cutaneous tissues.

**Figure 3:**
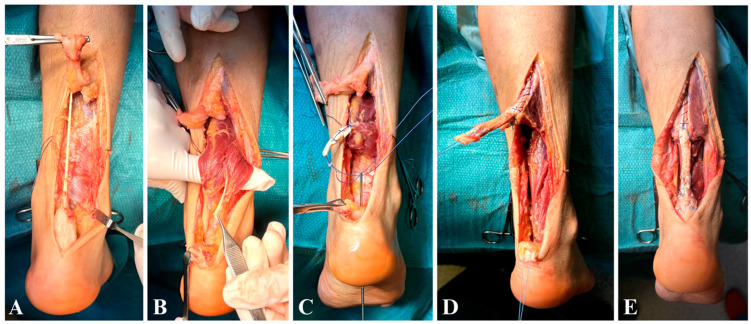
(**A**–**E**) right side: (**A**) intact plantaris tendon within the scarring tissue; (**B**) FHL tendon appears to be half in caliber compared to the FDL; (**C**) fixation of the combined FDL-FHL 1 cm anterior to the native Achilles stump; (**D**,**E**) turndown flap with no prominent knots.

- **Right Side:** The plantaris tendon was sacrificed as it was severely attenuated and deemed unsuitable for augmentation (Figure 3A). Initially, the FHL tendon was harvested for transfer; however, intraoperative evaluation revealed that its caliber was insufficient to adequately reconstruct the defect (Figure 3B). Consequently, the FDL tendon, which was nearly double the size, was also harvested and combined with the FHL for transfer (Figure 3C). The tendons were fixated 1 cm anteriorly to the native Achilles stump in a 5.5 mm calcaneal tunnel with a 7 × 23 mm biocomposite interference screw (MILAGRO™ Advance Interference Screw). The reconstruction was augmented with a turndown flap (Figure 3D) to reinforce the repair (Figure 3E).

Tendon sutures were placed with knots buried beneath the surface (subcutaneous technique) to prevent prominence under the skin. Terminal-to-terminal sutures for the turndown flap were performed with a locking Krackow technique using a heavy braided non-absorbable no.2 Tycron suture, and two stay stitches were added to prevent distal propagation of the flap. The peritenon was isolated and sutured over the reconstructed tendon using a continuous 3-0 Monocryl suture to ensure complete coverage.

**Figure 4:**
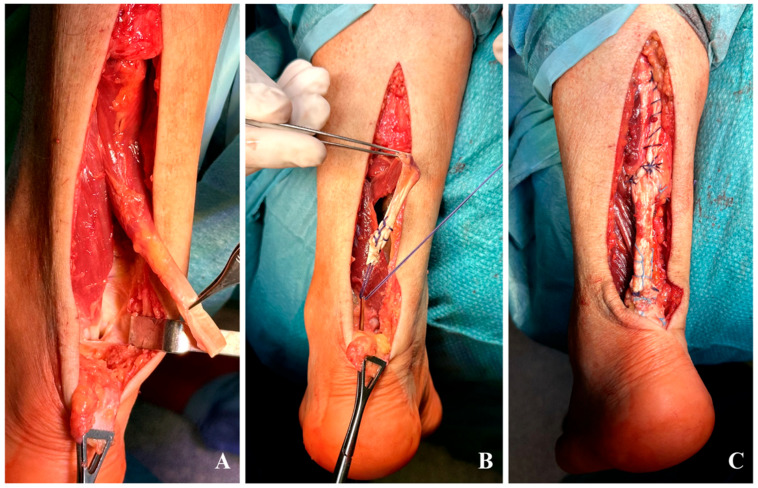
(**A**–**C**) left side: (**A**) FDL tendon appears to be much larger compared to the FHL; (**B**) fixation of the FDL 1 cm anterior to the native Achilles stump; (**C**) turndown flap with no prominent knots.

- **Left Side:** Based on the intraoperative findings from the right side, the FDL tendon was directly selected for transfer due to its larger caliber (Figure 4A), and fixated 1 cm anterior to the native Achilles stump in a 4.5 mm calcaneal tunnel with a 7 × 23 mm interference screw (Figure 4B). A turndown flap was used to augment and strengthen the repair (Figure 4C).

**Figure 5:**
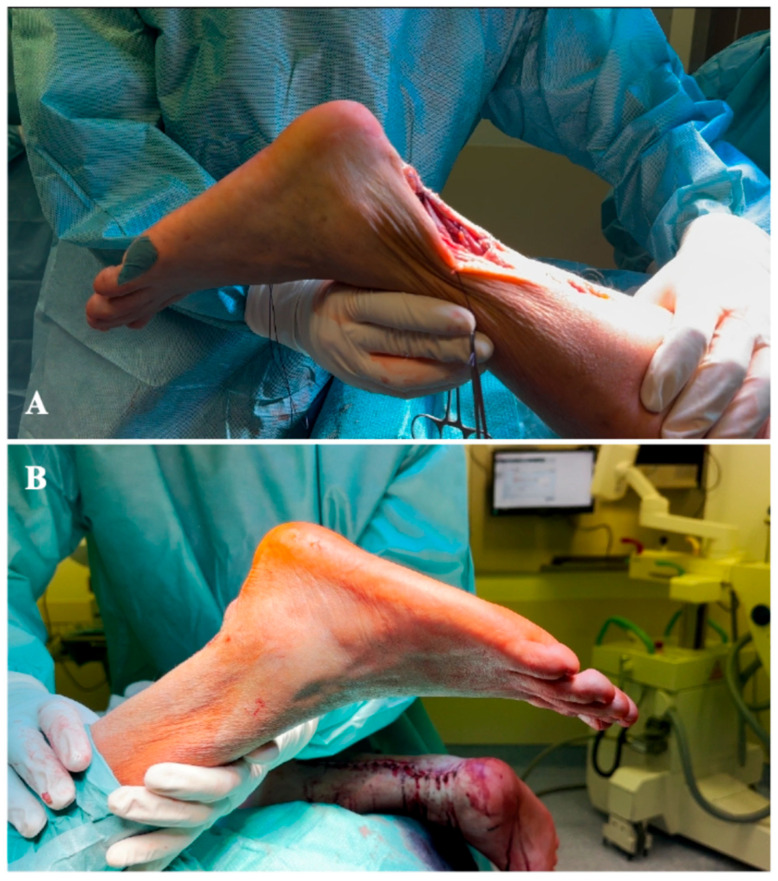
Resting ankle plantarflexion at 30 degrees of knee flexion after Achilles reconstruction on the right side (**A**) and on the left side (**B**).

Both procedures involved meticulous dissection to protect neurovascular structures. The tension aimed to balance functional restoration and avoid over-tightening, but since we didn’t have a healthy contralateral side for comparison, we recreated a resting plantarflexion of 15–20 degrees bilaterally while maintaining the knee flexed to 30 degrees (Figure 5).

### 2.4. Postoperative Management

Postoperatively, the patient was immobilized in bilateral equinus casts with strict non-weight-bearing precautions for six weeks with Low-Molecular-Weight Heparin (LMWH) prophylaxis. After cast removal, the patient transitioned into Controlled Ankle Motion (CAM) boots equipped with five heel wedges to progressively restore the neutral ankle position. These wedges were removed sequentially, one wedge every ten days, reaching a neutral (90-degree) position at approximately 12 weeks postoperatively.

**Figure 6:**
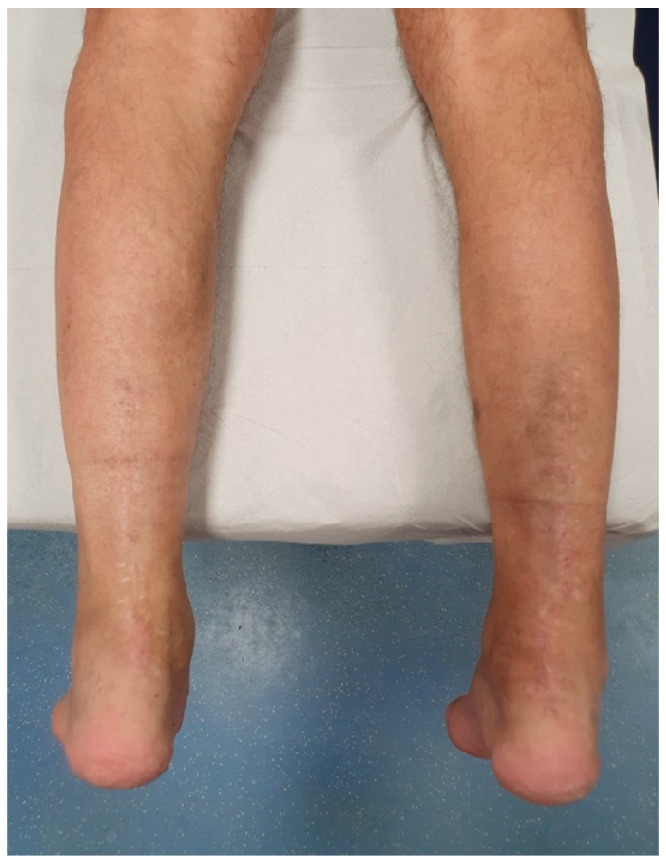
Complete wound healing at 3-month follow-up.

Wound care was managed with weekly dressing changes, and sutures were removed at three weeks. At the three-month follow-up, the CAM boots were discontinued (Figure 6), and the patient began full weight-bearing with crutches. Physical therapy was intensified with hydrokinesiotherapy, aimed at enhancing range of motion (ROM) and normalizing gait patterns. Once independent ambulation without walking aids was confidently established, rehabilitation transitioned to dry-land exercises, specifically designed for muscle strengthening and functional recovery.

**Figure 7:**
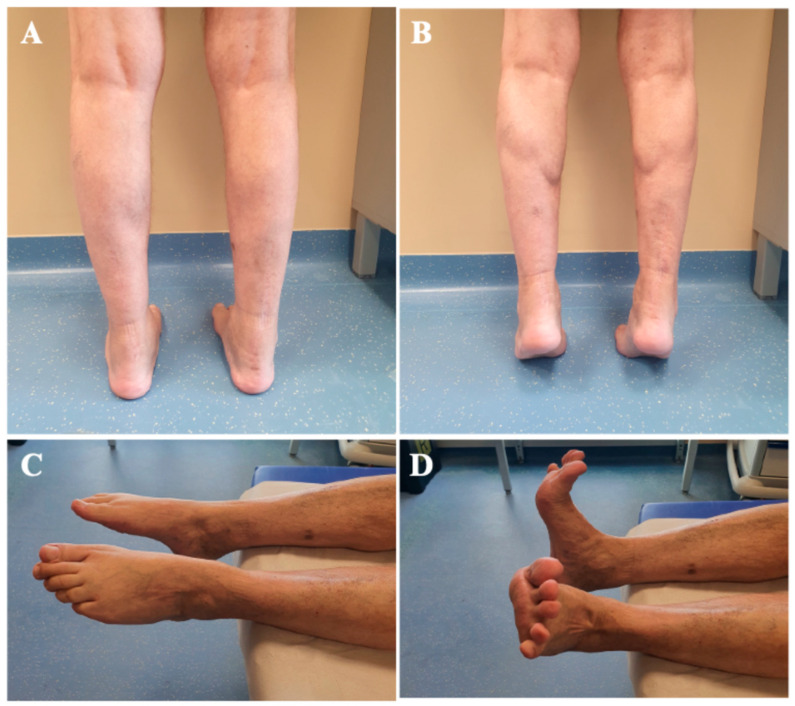
Clinical evaluation at six-month follow-up demonstrating the patient’s ability to stand on tiptoes, showing a good gastrocnemius muscle belly profile (**A**,**B**), and complete bilateral ankle range of motion (**C**,**D**).

By six months, the patient had resumed full-time occupational duties, demonstrating significant functional improvement with an AOFAS score increased to 85. At this point, ankle ROM was fully restored bilaterally, and the patient reported minimal residual morbidity attributable to tendon transfer procedures. Despite the bilateral harvest of the FDL, the patient exhibited 5/5 muscle power in lesser-toe plantar flexion, with no reports of balance disturbances or gait impairment. On the right side, where the FHL was also harvested, while a loss of active flexion at the hallux interphalangeal joint is expected, the patient demonstrated no functional instability or push-off weakness during normal walking. He was capable of tiptoe walking without difficulty, noting only a slightly reduced calf strength on the left side. Clinical evaluation revealed a well-defined gastrocnemius muscle profile, suggesting substantial recovery of muscle trophism (Figure 7). This outcome supports the decision to preserve the muscle belly through the use of turndown flap augmentation. A supplementary video documenting the patient walking on tiptoes is provided as additional supporting material (Video S1).

**Figure 8:**
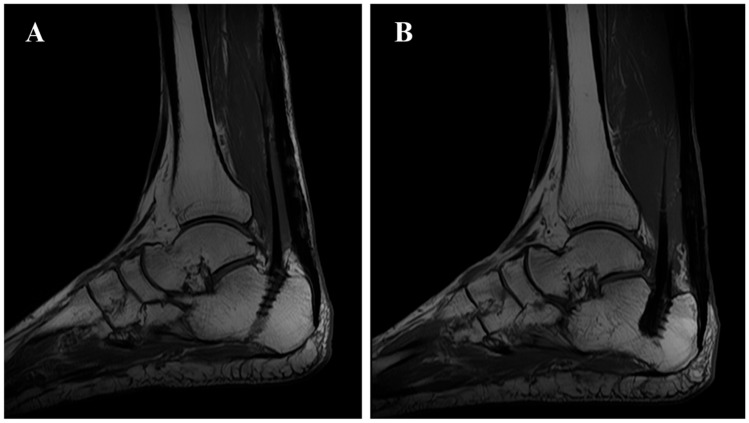
T1-weighted MRI at 12 months revealing full Achilles tendon continuity, with the transfer in place with tendon hypertrophy at the stump ((**A**) left foot; (**B**) right foot).

The 12-month follow-up included Magnetic Resonance Imaging (MRI) control (Figure 8). These images revealed full Achilles tendon continuity, demonstrating successful integration of the reconstruction. The tendon transfers (combined FDL and FHL on the right side, and FDL on the left side) were observed to be in place and correctly inserted at the calcaneal level. This insertion was situated approximately 1 cm anteriorly to the native Achilles stump. Notably, the MRI T1 images also showed tendon hypertrophy at the stump, confirming insertional tendon hypertrophy of the transfer.

## 3. Discussion

Chronic Achilles tendon ruptures are among the most challenging injuries to repair due to the large defects, tendon retraction, and chronic degeneration often associated with these cases. The complexity of these repairs necessitates the use of advanced techniques such as tendon transfers and biological or artificial augmentations to restore function and optimize outcomes. While several options exist, including V-Y advancements, turndown flaps, and various tendon transfers (FHL, FDL, or PB), the optimal management strategy for large gaps remains a subject of ongoing debate.

Among surgical options, flexor hallucis longus (FHL) transfer is considered the gold standard due to its biomechanical advantages, proximity, and vascular supply. FHL transfer provides reliable strength and promotes vascularization of the reconstructed tendon, though it can result in minimal loss of strength at the hallux interphalangeal joint without significantly affecting gait [4]. FHL tendon transfer can be isolated or combined biological augmentations, such as V-Y advancement or turndown flaps, as they help restore tendon length and improve biomechanical properties. Both methods have shown significant improvements in functional outcomes, pain reduction, and patient satisfaction [5]. While FHL transfer alone requires less operative time, the combined approach offers superior fixation strength and slightly higher functional scores [6].

While the 2024 evidence-based guidelines by Feng et al. primarily recommend FHL or Peroneus Brevis (PB) transfers for gaps between 3 and 6 cm, they do not explicitly list FDL transfer as a primary indication [7]. Nevertheless, FDL transfer is recognized as a documented operative alternative that provides good clinical outcomes with low complications and donor site morbidity [8].

Comparing our approach with other local and free grafts is essential for contextualizing the surgical choice. The Peroneus Brevis (PB) transfer is a suitable option for gaps under 6 cm but is associated with a statistically significant reduction in ankle eversion strength. For larger gaps exceeding 6 cm, such as the 6.5–7 cm defects in our patient, current guidelines often suggest free tendon autografts. While free gracilis or semitendinosus autografts provide excellent mechanical bridging, their harvest can be surgically demanding. Specifically, in a single-stage bilateral reconstruction, utilizing hamstring autografts would have required a change in the patient’s intraoperative positioning, increasing the technical complexity and prolonging the cumulative operative time. Furthermore, the decision to utilize a tendon transfer rather than a passive free graft was based on the functional status of the gastrocnemius-soleus complex. In long-standing ruptures, irreversible muscle atrophy and fatty infiltration often compromise the contractile potential of the original muscle after 3 to 6 months. While a free graft acts merely as a bridge, a local tendon transfer introduces a fresh, functional “motor unit” to actively drive plantarflexion. This is particularly advantageous when the viability of the gastrocnemius is uncertain [7].

The timing of intervention is another critical factor. Muscle atrophy due to disuse becomes increasingly irreversible as structural changes like loss of muscle fibers and fibrosis set in. Early surgical intervention within this window with techniques such as V-Y advancements and turndown flaps are particularly effective in reconnecting muscle to tendon optimizes the potential for muscle revitalization and functional recovery, thereby preventing further atrophy of the gastrocnemius and soleus muscles.

### Literature Review

To our knowledge, only four case reports in the literature provide insights into the management of chronic bilateral Achilles ruptures:

Zhang et al. (2020) [9] described isolated modified FHL transfers augmented with the plantaris tendon performed in two separate surgeries. The postoperative protocol included four weeks of immobilization, followed by partial weight-bearing and progressive physical therapy [9]. FHL tendon transfer is a reliable and biomechanically advantageous option due to its proximity, synchronized function with the Achilles during gait cycle, and strong vascular supply promoting vascularization of the reconstructed tendon. It is also the functionally strongest tendon in the calf, following the triceps. It is generally the preferred option for tendon transfer. This results in loss of strength at the hallux interphalangeal joint with no relevant functional gait impairment [4].

In contrast, both Polidoro et al. (2020) [10] and Dhillon et al. (2010) [11] reported the use of LARS ligament reconstruction in managing bilateral Achilles tendon ruptures. Polidoro described excellent functional outcomes with early mobilization and weight-bearing two weeks postoperatively, emphasizing the advantages of this technique in facilitating early recovery. However, Dhillon highlighted significant complications, including bilateral wound dehiscence necessitating flap coverage and debridement, underscoring the risks of using synthetic ligaments in poorly vascularized regions. Synthetic ligament augmentation, such as LARS, provides high initial strength while avoiding graft related morbidity, but carries risks of infection, wound related problems and impaired proprioception [10,11,12].

Katakura et al. (2021) [13] utilized turndown flaps augmented with artificial ligament and plantaris tendon in a single-stage bilateral surgery. Postoperative care involved immediate weight-bearing with crutches wearing patellar tendon bearing braces with heel wedges for 5 weeks, followed by removal of 1 wedge per week for 4 weeks [13].

These cases underscore the variability in surgical techniques and highlight the importance of tailoring approaches to patient-specific anatomical and functional needs. Each technique’s pros and cons offer insights into balancing repair strength, rehabilitation, and surgical complexity [14,15].

In our case, we opted for simultaneous bilateral reconstruction in a single-stage procedure. This approach offered the advantage of reducing cumulative anesthesia exposure and streamlining the overall rehabilitation process. Although performing bilateral surgery simultaneously poses challenges, particularly regarding postoperative mobility and weight-bearing restrictions, our decision was strongly influenced by the patient’s general health status, motivation, and robust support system, which facilitated compliance and recovery.

Secondly, corticosteroid therapy was gradually tapered in coordination with the rheumatology team in the weeks preceding surgery, with complete discontinuation immediately after the operation. Given that corticosteroids inhibit tenocyte proliferation and impair collagen cross-linking, tapering therapy aimed to mitigate potential risks such as impaired wound healing and increased infection rates. Although literature specifically addressing the effectiveness of corticosteroid tapering in this context is limited, this strategy represents a cautious approach to optimizing surgical outcomes in patients with rheumatoid arthritis undergoing tendon reconstruction. Future studies are needed to better define the precise clinical benefits of this approach.

Finally, regarding tendon transfer selection, we employed a flexor digitorum longus (FDL) transfer based on intraoperative assessment. While the FHL tendon transfer is more commonly described in literature due to its biomechanical alignment and reliability, the FDL transfer has emerged as a valid alternative, providing comparable functional results with potentially reduced donor-site morbidity, minimizing complications associated with reduced strength at the hallux during the push-off phase of gait. In our case, the initial attempt to use FHL on the right side demonstrated inadequate tendon caliber, prompting the intraoperative decision to combine it with the larger FDL tendon. Based on this experience, we directly selected FDL for the reconstruction of the left side. This highlights the importance of intraoperative flexibility when anatomical variations limit gold-standard options. Relying solely on the strength of the FDL might not be enough to compensate for gastrocnemius-soleus function, therefore a turndown flap augmentation is advised when using this transfer, with the potential benefit of revitalizing the detached muscle belly [8].

## 4. Conclusions

This case underscores the feasibility of simultaneous bilateral Achilles tendon reconstruction in chronic rupture cases, particularly in patients with systemic conditions like rheumatoid arthritis. A combined approach using FHL and FDL transfers with bilateral turndown flap augmentation provided excellent functional outcomes. The integration of biological augmentation techniques, such as turndown flaps, enhances repair strength and durability in cases of significant tissue loss. Future studies should focus on long-term comparisons of various reconstruction techniques and the role of augmentation in chronic Achilles ruptures.

## Data Availability

The original contributions presented in this study are included in the article/Supplementary Material. Further inquiries can be directed to the corresponding author.

## Supplementary Materials

The following supporting information can be downloaded at: https://www.mdpi.com/article/10.3390/jcm15030922/s1, Video S1: tip toe walking at 6 months.

## Abbreviations

The following abbreviations are used in this manuscript:

AOFAS	American Orthopaedic Foot & Ankle Society
LMWH	Low-Molecular-Weight Heparin
CAM	Controlled Ankle Motion
DVT	Deep Vein Thrombosis
FDL	Flexor Digitorum Longus
FHL	Flexor Hallucis Longus
LARS	Ligament Augmentation and Reconstruction System
MRI	Magnetic Resonance Imaging
PB	Peroneus Brevis
RA	Rheumatoid Arthritis
ROM	Range of Motion

## References

[1] Habusta S.F. (1995). Bilateral Simultaneous Rupture of the Achilles Tendon: A Rare Traumatic Injury. Clin. Orthop. Relat. Res..

[2] Khanzada Z., Rethnam U., Widdowson D., Mirza A. (2011). Bilateral spontaneous non-traumatic rupture of the Achilles tendon: A case report. J. Med Case Rep..

[3] Baruah D.R. (1984). Bilateral spontaneous rupture of the Achilles tendons in a patient on long-term systemic steroid therapy. Br. J. Sports Med..

[4] Den Hartog B.D. (2003). Flexor Hallucis Longus Transfer for Chronic Achilles Tendonosis. Foot Ankle Int..

[5] Koh D., Lim J., Chen J.Y., Singh I.R., Koo K. (2019). Flexor hallucis longus transfer versus turndown flaps augmented with flexor hallucis longus transfer in the repair of chronic Achilles tendon rupture. Foot Ankle Surg..

[6] Apinun J., Jenvorapoj S., Arirachakaran A., Kongtharvonskul J. (2020). Clinical outcomes of chronic Achilles tendon rupture treated with flexor hallucis longus grafting and flexor hallucis longus grafting plus additional augmentation: A meta-analysis. Foot Ankle Surg..

[7] Feng S.-M., Maffulli N., Oliva F., Saxena A., Hao Y.-F., Hua Y.-H., Xu H.-L., Tao X., Xu W., Migliorini F. (2024). Surgical management of chronic Achilles tendon rupture: Evidence-based guidelines. J. Orthop. Surg. Res..

[8] de Cesar Netto C., Chinanuvathana A., da Fonseca L.F., Dein E.J., Tan E.W., Schon L.C. (2019). Outcomes of flexor digitorum longus (FDL) tendon transfer in the treatment of Achilles tendon disorders. Foot Ankle Surg..

[9] Zhang X., Ruan F., Wu Y., Lu H. (2020). Chronic bilateral asynchronous achilles tendon rupture treated using modified whole flexor hallucis longus transfer reconstruction. Medicine.

[10] Polidoro F., Rea R., Fascione F., Salini V., Belluati A. (2020). Neglected complete bilateral Achilles tendon rupture. Clinical case presentation, treatment and follow-up. Acta Bio Medica Atenei Parm..

[11] Dhillon M.S., Chauhan D., Kumar V., Saini U.C. (2010). Reconstruction of bilateral spontaneous chronic idiopathic Achilles tendon rupture using LARS ligament: Case report. Foot.

[12] Ibrahim S.A.R. (2009). Surgical treatment of chronic Achilles tendon rupture. J. Foot Ankle Surg..

[13] Katakura M., Jujo Y., Okugura K., Mori Y., Hayashi K., Koga H., Takao M. (2021). Simultaneous reconstruction of the bilateral chronic achilles tendon rupture with early functional rehabilitation: A case report. Foot Ankle Surg. Tech. Rep. Cases.

[14] Eken G., Misir A., Tangay C., Atici T., Demirhan N., Sener N. (2021). Effect of muscle atrophy and fatty infiltration on mid-term clinical, and functional outcomes after Achilles tendon repair. Foot Ankle Surg..

[15] Xu Y., Li C., Liu T., Xiang F., Deng Y., Li Z., Wei D. (2023). Long-term outcome of flexor hallucis longus tendon transfer for chronic Achilles tendon rupture with large defect: A retrospective series. Medicine.

